# Diabetes Care Disparities in Deaf/Hard of Hearing and Blind/Low Vision Populations

**DOI:** 10.1007/s11892-024-01565-z

**Published:** 2024-12-30

**Authors:** Allyson S. Hughes, Karissa Mirus, Nazanin M. Heydarian, Michelle L. Litchman

**Affiliations:** 1 Ohio University Heritage College of Osteopathic Medicine, Athens, OH USA; 2 College of Nursing, University of Utah, 10 South 2000 East, Salt Lake City, UT 84112 USA; 3 University of Texas at Rio Grande Valley, Edinburgh, TX USA

**Keywords:** Diabetes, Deaf, Heard of hearing, Blind, Low-vision, Sign language, Accessibility

## Abstract

**Purpose of Review:**

Describe the connection between Deaf/hard of hearing (DHH) and diabetes, explain the bidirectional relationship of blind/low vision (BLV) and diabetes, characterize challenges DHH and BLV populations face when seeking healthcare regarding their diabetes management. Highlight the inaccessibility of diabetes technology in these populations. Provide best practices when communicating with DHH and BLV people in the clinical setting.

**Recent Findings:**

Diabetes disparities exist in DHH and BLV populations due to systemic barriers to health equity related to access and communication. Structural barriers, risk factors, social determinants of health, and the U.S. healthcare system do not support the DHH and BLV communities. Importantly, healthcare professionals do not receive adequate training on communication and treatment of DHH and BLV populations. Together, social determinants of health, such as healthcare access and quality, education access and quality, and lack of adequate clinician training allow ableism to persist and drive health disparities in these communities.

**Summary:**

Health disparities faced by DHH and BLV populations are driven by barriers to diabetes standards of care. These inequities must be rectified to improve and maintain high quality care.

## Introduction

Globally, 1 in 6 people are living with a disability [1]. People with diabetes experience disability at higher rates due to diabetes-related complications [2]. People with diabetes are twice as likely to become Deaf/hard of hearing (DHH) compared to the general population. Moreover, there is a bidirectional relationship between diabetes and vision loss such that people who are blind/low vision (BLV) are more likely to develop type 2 diabetes and people who have type 2 diabetes are more likely to experience vision loss [3, 4]. Diabetes is complex, and when someone is managing diabetes while living with BLV and/or DHH, tailored approaches to providing clinical care are critical. In addition, education and technology designed to support people in self-managing their diabetes is fraught with assumptions of “ablebodiedness”—the absence of disabilities. This paper will emphasize the diabetes care accessibility needs of the DHH or BLV communities. Unless otherwise indicated, the term diabetes is used to reference both type 1 and type 2 diabetes.

### Deaf/Hard of Hearing and Diabetes

There are 11 million DHH people in the United States who experience diabetes at higher rates than their hearing counterparts [5]. An estimated 500,000 DHH people use American Sign Language (ASL) [6]. According to a recent analysis of the National Health and Nutrition Examination Survey, DHH people are 3.2 times more likely to be diagnosed with diabetes and 1.6 times more likely to be at risk for diabetes compared to hearing people [7]. Importantly, rates of diabetes among Deaf middle-age and older adults who use ASL is higher (about 26%) than the general population (between 14 and 21%) for this age group [8]. This disparity is believed to be related to the limited availability of health information in ASL [9]. For example, information specific to diabetes risk, management of prediabetes, conditions where both diabetes and being deaf can co-occur is not readily available in ASL. Moreover, few clinicians are bilingual in English and ASL and there are even fewer Deaf clinicians who can create health materials in ASL, resulting in a dearth of health information in ASL. The major diabetes professional organizations have not developed materials in ASL despite doing so for other languages (e.g., Spanish). Among other chronic conditions, evidence suggests maladaptive childhood communication experiences (when a parent is unable to communicate with their DHH child in ASL, (see language deprivation described further in the health literacy section)) are associated with diabetes in DHH people [10].

People with diabetes are two times more likely to experience low- and high-frequency hearing loss when compared to people without diabetes [11]. While the etiology of coexisting diabetes with DHH is unknown, for those with existing diabetes, a higher A1c over prolonged periods of time [12] vascular disease, and neuropathy [13] have been linked to DHH. The American Diabetes Association’s Standards of Care recommend an audiology exam, if indicated, [14] however hearing is not prioritized and assessed routinely at diabetes clinical visits.

Genetic causes for both diabetes and DHH exist. Among all cases of diabetes, it is estimated that 0.5–2.8% are maternally inherited diabetes and deafness (MIDD) [15]. MIDD is caused by a m.A3243 > G mitochondrial DNA mutation and is transmitted via maternal inheritance. MIDD is related to: 1) maternal heritability of diabetes or impaired glucose with normal body mass index, 2) hearing impairment, and 3) maculopathy. Nearly half of patients with MIDD require insulin within 10 years of diabetes diagnosis [16]. Other associated conditions include stroke-like episodes, myopathy, encephalopathy, seizure, migraine and cognitive impairment. In addition to MIDD, DHH can also experience type 1 diabetes, type 2 diabetes, and other less common forms of diabetes, such as Wolfram Syndrome, Rogers Syndrome, and Hermann Syndrome [17].

### Blind/Low Vision and Diabetes

BLV are increasingly common in the United States, such that approximately 7.08 million people have low-vision and 1.08 million of those individuals are blind [18]. BLV occurs on a spectrum such that some can see changes in light, while others may rely on one eye for their vision. Rates of BLV are anticipated to increase due to an aging population coupled with increasing prevalence of type 2 diabetes [19, 20]. People with BLV frequently experience health disparities, particularly those over age 65 [2, 21]. Diabetes-related retinopathy is the leading cause of blindness in the United States. One in four Americans ages 40 and over have diabetes-related retinopathy [22]. Diabetes-related retinopathy is a major risk factor for the progression of other eye disorders that lead to BLV (e.g., cataracts, glaucoma, and macular degeneration) [23]. People who are blind are twice as likely to be diagnosed with diabetes as non-disabled people, [4, 21] and have an elevated rate of diabetes-related mortality compared to non-disabled persons [24].

Individuals with BLV often encounter substantial barriers to accessing quality healthcare, including communication issues with healthcare professionals (HCPs) and staff, lack of transportation, and inaccessible built environments and physical spaces [25]. People with diabetes and BLV also experience significant stigma and discrimination that interfere with their access to quality healthcare. For example, Heydarian et al. [21] reported a participant’s experience where their healthcare team would not reconnect their insulin pump following surgery because of assumptions of the participant’s incompetence in diabetes self-management. This study found HCPs stereotypes regarding BLV incompetence may lead them to treat BLV patients condescendingly and dismiss their reason for pursuing healthcare. Most participants reported being treated as if they were incompetent by their HCPs. There are also notable disparities in diabetes management self-efficacy among BLV individuals [26]. Importantly, a recent study of 181 BLV people found that Hispanic and Latino ethnicity was correlated with the type of glucagon prescribed, greater likelihood of DKA history, greater frequency of using blindness-related assistive technology, and lower likelihood of braille usage. Importantly, lower A1c was correlated with higher frequency of blindness-related assistive technology, using a white cane, and having a guide dog [27].

### Health Literacy in the Deaf/Hard of Hearing Community

Among the DHH population, low health literacy is common, even among DHH who are college educated [28]. Compared to hearing counterparts, DHH adults are 6.9 times more likely to have inadequate health literacy [29]. Surprisingly, when DHH were college educated, they are 14.6 times more likely to have lower health literacy levels than hearing people with significantly less education (high school or less) [29]. DHH individuals are born to hearing parents 90% of the time [30] who often do not learn ASL [31, 32] can result in ‘dinner table syndrome.’ Dinner table syndrome describes the habitual occurrence that takes place during mealtimes when DHH individuals are left out of conversations by hearing family members and friends, which results in low health literacy, social isolation, and language deprivation [33, 34]. As such, having hearing parents as a DHH person is considered a social determinant of health [35].

DHH individuals attend Deaf institutions (taught in ASL) and mainstream schools (taught in English with or without an ASL interpreter). Lack of communication in ASL within a school system, via direct communication or ASL interpreter, can impact learning negatively. This lack of education access and quality can impact future job prospects and income level. A scoping review identified food insecurity was linked to income level, mental health status, and caregiver communication [36]. Decades of diabetes research indicate low-income level is correlated with higher A1c and diabetes health outcomes [37].

### Health Literacy in the Blind/Low Vision Community

Health literacy is rarely studied in the BLV community. The lack of health literacy research for this community is troubling because decades of research have established that health literacy impacts the relationship between HCP and patient and health outcomes [38, 39]. Low health literacy has been detected in the few studies that have been conducted in this community. In a study of Danish people with type 1 diabetes who had vision loss from diabetes, participants had a significantly lower level of health literacy regarding finding and understanding health information [40]. In a secondary data analysis of qualitative data, participants stated barriers to gaining new information in an accessible format and issues with HCP viewpoints [41]. Importantly, BLV people do not have the same access to health information as their sighted peers due to the lack of accessibility [42]. BLV people attend institutions tailored to the BLV community (such as the Perkins school for the Blind in Massachusetts) which focuses on verbal and audio instruction and teaching braille. In social settings, BLV people may rely on descriptions of the environment from others.

### Communication Approaches in the Deaf/Hard of Hearing Community

DHH persons have a range of communication approaches. Sign language is the gold standard language for many DHH people. Sign language is a communication system comprised of hand gestures, body movements (e.g., shoulder shifts), and facial expressions. Importantly, sign language varies by region/country. For example, in the United States and parts of Canada, ASL is used. However, Indo-Pakastani Sign Language, Mexican Sign Language, British Sign Language and many other sign languages are used globally. Contrary to some beliefs, ASL is not visual English. ASL has its own grammatical structure and syntax. Some DHH individuals will be monolingual, knowing only ASL, while some will be bilingual, knowing ASL and another voiced/written language, such as English. While some DHH people have been trained to read lips, some data suggests about 30% of lipreading is accurately understood in optimal conditions (e.g., no accents, speech impediments, or facial hair covering the mouth; well-lit room, proximal distance is reasonable, etc.) [43].

Technoableism assumes technology can “fix” a disability and restore “normalcy” [44]. While some DHH individuals may benefit from hearing aids or cochlear implants, it should not be used as a sole factor to satisfy the communication required to understand critical information shared within the medical setting. In fact, a combination of different methodologies (e.g., medical notes transcribed in an appropriate vocabulary level, ASL interpreter, certified deaf interpreter, voice-to-text, etc.) should be considered to ensure that persons with significant hearing loss benefit from these exchanges. Importantly, a patient’s request for accessible services fall under the American Disabilities Act [45] and every effort should be made to grant their request. Unfortunately, a meta-analysis found that HCPs prefer to communicate verbally or with gestures, despite DHH desiring other forms of communication that better meet their needs. This misalignment with communication preferences can result in miscommunication [46]. Though video relay interpreting (VRI, a virtual ASL interpreter) can be used in medical appointments when an in-person ASL interpreter is not available, it is not the preferred method by DHH due to connectivity issues, the ASL interpreter not being medically trained, among other issues [47]. VRI should be used only in emergency situations or as a last resort [48]. Family and friends as proxy interpreters should not be used as they are not certified and may not fully interpret information correctly [48].

### Communication Approaches in the Blind/Low Vision Community

The BLV community employs a range of communication approaches including tactile, auditory, haptic, and visual enhancement tools to allow members of the blind community to access information that is predominantly offered in a visual format (e.g., written language, graphics). Braille is a tactile code analogous to written language. It uses a grid of six bumps where different combinations of raised bumps on the grid represent letters, numbers, punctuation symbols, and contractions. Braille contractions do not exist in the print counterpart (e.g., a single symbol will represent “-ch-” and “-th-”). There are similarities in overall prose comprehension between Braille and print readers [49, 50]. Differences have been found in word perception such that print readers process words as a single unit whereas Braille readers process words one character at a time [51–53]. Exploratory research links Braille literacy with more favorable outcomes pertaining to diabetes distress [54]. Braille literacy is mixed across research samples and our research has found 52%—62% Braille literacy. Additionally, A1c levels, disability duration, and ethnicity were found to be related to braille usage [27].

There are many tools to assist BLV individuals with communication. Tools like the Kurswell Reader and character recognition software to digitize the text in a format that can be read via smartphones. Phone apps exist that will digitize printed text. One of the most popular options are screen readers such as JAWS, TalkBack, or VoiceOver. There are also refreshable Braille displays to facilitate reading digital content. More recently, BLV people use voice-activated assistants such as Amazon Echo, Alexa and Siri. Smart phone apps such as Be My Eyes and AIRA connect the user with a sighted person who provides live audio description of what they are shown through the BLV person’s camera. Artificial intelligence-based technology such as Seeing AI and LookOut use artificial intelligence to provide audio descriptions of what the user points their camera at. There are also screen magnifiers that enlarge the content on the screen for people with low vision. On technology, such as computers, smart phones, and tables, settings are available to change contrast and brightness, and use color filters. There is emerging technology that is able to produce refreshable tactile graphics that may be helpful in communicating visuals such as glucose trends from CGM data.

### Healthcare Barriers Faced by the Deaf/Hard of Hearing Community

Being DHH is perceived differently by people who are themselves DHH and the medical community. DHH individuals who use ASL as the primary means for communication view being Deaf as a cultural heritage, with its own language, values, traditions, norms, and identity. Quite differently, HCP often refer to DHH as a pathological condition necessitating medical intervention [55]. This mismatch based in ableism can create challenges when DHH individuals seek medical care.

In healthcare settings, ASL interpreters are not provided half of the time, [56] including during diabetes specialty visits [57]. When an interpreter is provided, about one-third of the time the interpreter is not comfortable interpreting medical situations and therefore unqualified [58]. In another study, an ASL interpreter was only considered as a last resort [59]. Furthermore, Deaf individuals may not disclose important health information if the interpreter is not receptive to the Deaf individual and if the HCP is not using patient-centered communication behaviors [60]. Inability to communicate important information about a condition, such as diabetes, that requires multiple daily self-management tasks in a Deaf person’s primary language, ASL, can negatively impact diabetes outcomes [60]. Deaf Diabetes Can Together, a diabetes program guided by a DHH community advisory board, identified that the development of an ASL glossary of diabetes terms was necessary to improve communication between the DHH person with diabetes, ASL interpreter, and HCP [61].

DHH people experience a myriad of challenges when seeking healthcare due to social and informational marginalization. Diabetes is a complex condition requiring ongoing education and support that many DHH people do not receive. Evidence indicates about 60% of the time, Deaf people do not understand HCP instructions [62]. As such, Deaf people are turning to other Deaf people for health education within e-Health platforms, such as online communities. Deaf online communities are used to discuss health topics using short videos in sign language uploaded by users. In a sample of 515 Deaf adults, half used online communities and use was more common among those with comorbid conditions, such as diabetes [63]. This lack of access to high quality healthcare is a social determinant of health.

### Healthcare Barriers Faced by the Blind/Low Vision Community

People with BLV experience significant barriers with transportation, cost, inaccessible physical environment, lack of assistive technology and rehabilitation devices, and negative attitudes towards people with disabilities. Specific to diabetes, there is a dearth of information regarding clinical barriers to care in the BLV population. Importantly, disability competence—sensitivity and responsiveness to disability culture and etiquette—or lack thereof, is critical for supporting diabetes self-management [21]. In negative experiences interacting with HCPs, BLV people reported that the HCP treated them in a way that was inconsistent with blindness etiquette (e.g., giving print health information materials to blind patients, using inappropriate methods such as pulling a patient’s white cane to lead them around the clinic) [21]. Culturally incompetent communication and behavior can undermine self-management and health-care-seeking behavior by way of patient mistrust [64].

### Diabetes Technologies

Diabetes technologies have been built for hearing and sighted populations in mind. This lack of built environment, a social determinant of health, specific to DHH and BLV populations reduces access to the American Diabetes Association Standards of Care. Despite their high technology adaptation, DHH and BLV populations face barriers to gaining access to the latest diabetes technology. This inequity increases the magnitude of health disparities for DHH and BLV populations. Generally, DHH and BLV people have high technology adoption from using a variety of devices for daily living to communicate, even among older adults [65]. Below we review a variety of diabetes technologies in the context of DHH and BLV considerations.

### Insulin Pumps and Continuous Glucose Monitoring

Insulin pumps are used to continuously deliver insulin. Insulin pump alarms alert people with diabetes when the insulin pump battery level is low, if the insulin reservoir levels are low, when insulin delivery is occluded, and several other events. Continuous glucose monitors (CGM) are used to monitor glucose levels on an ongoing basis through displayed data on a receiver or smart phone. CGM alerts to out-of-range glucose levels or sensor errors (e.g., sensor not working, transmitter not in range). HCPs will need to ensure the proper amount of training a DHH/BLV person might need with CGM and insulin pumps. For example, extra time is needed for DHH when using an ASL interpreter to allow for the interpretation process. Preparing ASL interpreters ahead of time with any literature on diabetes technology language can also help improve the flow of communication. HCPs can also consider a normal saline insulin pump trial to ensure a specific insulin pump will work for individual DHH/BLV patients during the training period.

While BLV and hard of hearing individuals may be able to use audible alarms, many DHH people cannot. Ideally, accessible technologies provide a variety of alert options, including audio, haptic (e.g., vibrations), and visual (e.g., flashing lights) to ensure accessibility for all populations. See Table 1 for insulin pump and CGM device specific alarms in the context of accessibility. BLV populations often rely on screen readers to access information on digital devices, such as smart phones. However, the majority of diabetes technology apps are not screen reader friendly, furthering disparities in diabetes technology use in BLV populations.

**Table 1:** Insulin Pump and Continuous Glucose Monitor Accessibility

	**Audible Alarm**	**Haptic Alarm**	**Visual Alarm**
**Insulin Pumps**			
Medtronic 670 g	X	X	
Medtronic 770 g	X	X	X^a^
Medtronic 780 g	X	X	X^a^
Tandem X2	X	X	X^a^
Tandem Mobi	X	X	X^a^
Omnipod Eros	X	X^b^	
Omnipod DASH	X	X^b^	
Omnipod 5	X	X	X^a^
**Continuous Glucose Monitoring**			
Dexcom G6	X	X	X^a^
Dexcom G7	X	X	X^a^
Freestyle Libre 14 day	X		
Freestyle Libre 2	X		X^a^
Freestyle Libre 3	X	X	X
Eversense		X	
Eversense XL		X^a,c^	
Medtronic Guardian Connect	X	X	X^a^

^a^Diabetes technology mobile application must be connected to compatible smartphone and flash notification activated in accessibility alerts

^b^Personal Diabetes Manager (PDM) only, not the pod

^c^On body vibration at the implantation site through the transmitter

Often devices rely on “hacks” to make them more accessible, however, hacks are not accessibility. External tools, such as Sugar Pixel [66] and iLuv Smart Shaker [67] can also be used to support vibration alarms for some CGM devices and serve as accessibility tools for the DHH community. Moreso, these additional external tools are not covered by insurance, creating another barrier to device adoption. These external tools are not equivalent to designing a product with accessibility in mind and do not replace the user-friendliness of a product designed to be accessible “out of the box”. Ideally, diabetes technology would be 100% accessible for both DHH and BLV populations without external tools.

The most pressing need to make diabetes technology accessible for BLV people is to have all features available entirely on an accessible phone app. While looping is accessible by phone app, it has not been studied in the BLV community and may have other barriers in the initial technology set-up. Traditional insulin pump screens serve as one of the biggest accessibility barriers for BLV people with diabetes. CGM devices, such as Dexcom and Freestyle Libre, can be connected to other devices (e.g. smart phone, smart watch) that allow for vibratory alarms and use of built-in screen readers to access the information projected to the mobile app. The most recent Dexcom G7 software update allows the sensor to be paired directly with an apple watch, which may improve accessibility for both DHH and BLV. All diabetes technology should have voice-output features to announce readings and be compatible with screen readers. Training for diabetes technology should be available not only online but also in braille and audio. Importantly, current insulin pumps and CGMs have contraindications for DHH and BLV people. It is critical to incorporate universal design principles and engage with these communities in the design and testing of diabetes technology before it is available on the market. Overall, addressing these needs can significantly improve the independence and quality of life for DHH and BLV individuals managing diabetes, ensuring they have equal access to life-saving technology.

### Best Practices Recommendations

Clinical best practices that meet the needs of DHH and BLV people with diabetes are important to facilitate communication, autonomy, and respect [68]. Importantly, diabetes self-management behaviors are achieved in a social context. Care partners (family, friend) provide a variety of helpful and harmful behaviors related to diabetes [64]. Care partners of DHH may have mixed hearing status (deaf, hard of hearing, hearing) and care partners of BLV may have mixed vision status (blind, low vision, sighted). Involved DHH care partners desire diabetes care that meets their cultural and linguistic needs [69] and yet, have fewer opportunities to support learning formally and informally [70]. For example, an ASL interpreter skilled in medical terminology is provided for diabetes education visits less than half of the time [57]. A DHH care partner may experience language barriers communicating with a knowledgeable hearing person. Reaching out to another person in the DHH community, who encounters the same access issues creates challenges in knowledge attainment [70]. To be helpful, and not harmful, a DHH care partner must have accurate diabetes information.

The American Diabetes Association emphasizes the importance of patient-centered care to focus on patient preferences, needs and values [71]. However, paternalism can offset patient-centered care. Paternalism can be seen in DHH-hearing families, in the clinical setting, and within diabetes technology companies. In DHH-hearing families, the hearing individual can broker sign language, [72] and influence decision making. Clinicians have historically treated DHH individuals without properly educating the DHH person about their health condition and without informed consent. As highlighted above, paternalism refers to the caregiver's dominant attitude towards the care recipient. This approach, often practiced by clinicians, involves making decisions on behalf of the patient or client with the assumption that it is in their best interest.

### Deaf/Hard of Hearing

If a deaf person requests an ASL interpreter, make sure you provide one for their appointment. It is important to accommodate to the deaf person’s need if they prefer an in-person ASL interpreter. When you have a deaf patient, make sure to look directly at the patient, speak clearly at a moderate pace, and make sure to allow the interpreter to finish and enough time for the patient to process the information and ask any questions they may have. Rephrase and repeat key points, and utilize the teach-back method to ensure understanding. This means asking the patient to explain the information back in their own words. If communication remains a challenge, consider having a Certified Deaf Interpreter (CDI) with a hearing ASL interpreter who are familiar with medical terms. CDIs provide language nuances specific to the DHH community and aim to deepen the understanding of the information for the DHH individual. Be direct, explain the information clearly and the potential consequences of not following instructions. You can bring visual aids like charts and demonstrations for enhanced understanding. Make sure that your front office staff knows to be patient with ASL interpreter during scheduling calls and ask the patient if they need an ASL interpreter, CDI, and/or communication real-time translation (CART) for their appointment. CART is for DHH people who may not know sign language and need transcription to communicate with others (largely used in clinical and education settings). These practices will ensure clear communication, improved patient understanding, and ultimately, better health outcomes.

### Blind/Low Vision

Accessible introductions are an easy way to introduce yourself to someone who is BLV. Begin by stating your name, your pronouns, and your role (e.g., First time meeting: “Hello. My name is [your name], my pronouns are [your pronouns], and I will be your physician.” Then, you can ask if the person would like a visual description. If they do, you can begin visually describing yourself. You can begin by including race and/or skin-tone (detail and depth dependent on you), gender (i.e. woman, man, person, etc.). You can then describe your hair color/length/style (detail dependent on you), and clothing. Lastly, describe your speaking accent. Additional best practices include: always addressing the person directly, always handing documents to the person directly, ask if the person would like your help and give specific directions such as there is a chair three feet behind you. Avoid vague direction such as “over there” and making visual gestures. Education for clinic staff in these best practices can also assist in promoting health equity in the BLV population. HCPs should seek to improve the knowledge of all clinic staff and remove structural barriers that occur in clinic.

## Conclusion

Although diabetes treatment has greatly improved over the last three decades, there is still room for improvement in accessibility for BLV and DHH populations. Diabetes technology companies should engage and collaborate with the DHH and BLV communities to make these technologies accessible. These communities are excluded from the development and testing of new products. DHH and BLV people are frequently excluded from clinical research, which limits how well diabetes devices can work for them. People in these populations need to be engaged in device and treatment development from the beginning. There is much to be improved regarding communication between HCPs and DHH and BLV people in the clinic setting. More equitable healthcare for DHH and BLV populations requires additional training for clinicians, clinic staff, diabetes technology companies and professional organizations that produce diabetes education content.

## Data Availability

No datasets were generated or analysed during the current study.
